# Effects of Luteolin-7-O-Glucoside on Intestinal Microbiota Dysbiosis and Drug Resistance Transmission Caused by *Raoultella ornithinolytica* B1645-1: Modulating the Composition of Intestinal Microbiota and Promoting the Transfer of *bla*_NDM-1_ Gene from Genus *Enterococcus* to *Lactobacillus* in Mice

**DOI:** 10.3390/microorganisms11102477

**Published:** 2023-10-02

**Authors:** Zhaomeng Wu, Ronghui Gou, Longhua Sha, Chunfang Yu, Lixue Meng, Zhixiong Jin

**Affiliations:** 1 Hubei Key Laboratory of Embryonic Stem Cell Research, School of Basic Medical Sciences, Hubei University of Medicine, Shiyan 442000, China; wuzhaomeng2833@163.com (Z.W.); grh302601@163.com (R.G.); slh2000215@126.com (L.S.); yuchunfang2008@hbmu.edu.cn (C.Y.); 2 Hubei Key Laboratory of Wudang Local Chinese Medicine Research, School of Pharmaceutical Sciences, Hubei University of Medicine, Shiyan 442000, China

**Keywords:** luteolin-7-O-glucoside, intestinal microbiota dysbiosis, *Raoultella ornithinolytica*, drug resistance transmission, *bla*_NDM-1_

## Abstract

*Raoultella ornithinolytica* is an *Enterobacteriaceae* bacterium that can infect both humans and animals, while luteolin-7-O-glucoside (IOG) is a flavonoid that has broad effects on the intestinal microbiota of healthy animals. However, current studies lack sufficient data on intestinal microbiota dysbiosis and drug resistance transmission caused by *R. ornithinolytica* and the possible role of IOG. In this study, BALB/c mice were infected with *R. ornithinolytica* carrying *bla*_NDM-1_ gene and treated with IOG (3 mg/kg·d and 6 mg/kg·d) to analyze the diversity of intestinal microbiota and the transfer of *bla*_NDM-1_ between bacteria. The findings indicated that *R. ornithinolytica* B1645-1 exhibited a significant ability to enhance the *Firmicutes*/*Bacteroidota* ratio and increase the relative abundance of *Lactobacillus* and *Bacillus* after 48 h, where as 6 mg/kg·d IOG had an opposite effect. Moreover, *R. ornithinolytica* B1645-1 facilitated the emergence of drug-resistant bacteria and promoted *bla*_NDM-1_ gene transfer in *Enterococcus*, *Escherichia*, *Klebsiella*, *Acinetobacter*, *Bacillus*, *Brevibacterium*, and *Lactobacillus*. *Enterococcus* was the predominant genus at 48 h. Surprisingly, 6 mg/kg·d IOG significantly inhibited the production of drug-resistant bacteria and promoted *bla*_NDM-1_ gene transfer from *Enterococcus* to *Lactobacillus* at 144 h. However, the role of *Lactobacillus* as a recipient for drug-resistant genes should be of more concern.

## 1. Introduction

Luteolin-7-O-glucoside (IOG) is a kind of flavonoid abundantly present in the flower buds, leaves, roots, and stems of *Lonicera japonica* Thunb, *Herbataraxaci*, *Forsythia*, *Polygonum cuspidatum*, *Ginger*, and other plants [1,2,3,4,5]. These herbs are mainly used for the treatment of exogenous wind heat, febrile diseases, sores and carbuncles, and some infectious diseases in traditional Chinese medicine [6]. Furthermore, modern pharmacological studies also show that herbal extracts containing IOG possess antibacterial, anti-inflammatory [7], hypolipidemic [8], anti-ischemic [9], and anti-leishmania activities [10].

Oral administration is arguably the most effective and easy means of drug delivery thatis widely recommended. Following ingestion, IOG is not destroyed by gastric acidand is mainly hydrolyzed by *β*-glucosidase of gastrointestinal mucosa or intestinal microbiota, such as *Enterococci*, *Lactobacilli*, *Bacteroides*, and *Bifidobacteria*, to release luteolin. The small intestine absorbs luteolin, which is subsequently re-secreted into the intestine via hepatic excretion [11]. Although *Enterococcus* is particularly active in the metabolism of IOG, it is likely that strictly anaerobic organisms, such as *Lactobacilli* and *Bifidobacteria*,are primarily responsible for the hydrolysis of glycoside in the small intestine [11]. The metabolism of flavonoids is both influenced by and influences the composition of the intestinal microbiota. Kondapalli NB et al. reported that IOG significantly increased the levels of *Lactobacillus* and *Bifidobacterium* in the intestinal microbiota of healthy rodents [12]. In vitro studies have shown the antibacterial activity of IOG against *Staphylococcus aureus*, *Streptococcus pneumoniae*, *Bacillus subtilis*, *Enterococci, Salmonella typhimurium*, and *Escherichia coli* [13,14]. Therefore, it is plausible to suggest that the metabolism and biological activity of IOG may also influence the intestinal microbiota in vivo.

The mammalian gut contains an extremely dense microbial community (>10^12^ bacteria/g) that has a significant impact on the host’s immune systems [15]. Inflammatory responses from the gut immune system or pathogens can lead to suppression of anaerobic microbiota (e.g., *Lactobacillus* and *Bifidobacterium*) and boost the colonization density of *Enterobacteria*, such as *Klebsiella pneumoniae* and *E. coli* [16]. Many *Lactobacillus* species and *Bifidobacterium* species are generally considered safe and are often utilized as probiotics to ameliorate memory deficits, brain neuron damage, glial activation, and fecal microbiota composition [17]. Anaerobic bacteria play a crucial role in regulating inflammation, as demonstrated by the attenuation of intestinal inflammation in breast-fed infants supplemented with *Bifidobacterium longum* subsp. *infantis* [18]. Intestinal inflammation also facilitates horizontal gene transfer among bacterial populations, primarily through persister cells formed by pathogenic bacteria [19], which can promote plasmid transfer up to 99% between different *Enterobacteriaceae* strains. *Enterobacteriaceae* have the ability to acquire, accumulate, and disseminate resistance genes via mobile genetic elements from intestinal microbiota [20,21]. The persistent emergence of drug-resistant *Enterobacteriaceae* results in escalating morbidity, mortality, and healthcare costs [22].

*Raoultella ornithinolytica* is a Gram-negative aerobic bacterium belonging to the *Enterobacteriaceae* family [23]. It is primarily found in aquatic environments, soil, insects, and fish. This bacterium has the ability to convert histidine to histamine, which can cause fish poisoning [24]. In humans, it can lead to infections of the digestive tract, urinary tract, and blood, especially in immunocompromised patients [23,25]. Although *R. ornithinolytica* has been found to have weak pathogenicity in some studies, there is clinical concern about the impact of this strain and its multi-drug resistant variants on patients [26].

Current research lacks sufficient data on intestinal microbiota dysbiosis and the transmission of drug resistance caused by *R. ornithinolytica*. The potential role that IOG can play in regulating these effects is intriguing. To bridge this gap, we established a mouse infection model carrying the *bla*_NDM-1_ gene encoding New Delhi Metallo-β-Lactamases-1 (NDM-1), a carbapenemase. The aim was to investigate the impact of *R. ornithinolytica* on the microbiota in mice and evaluate the regulatory capacity of IOG on intestinal microbiota.

## 2. Materials and Methods

### 2.1. Strain and Reagents

*R. ornithinolytica* B1645-1 strain was isolated from a renal transplant patient’s blood at the Sinopharm Dongfeng General Hospital (Shiyan, China). The strain contained a 149.44 kb plasmid (Accession No.: MK510953), which carried multiple *β*-lactamase genes (*bla*_NDM-1_, *bla*_CTX-M-9_, and *bla*_TEM-1_) [27].

IOG and imipenem (IPM) were purchased from Tianjin Vientiane Hengyuan Technology Co., Ltd. (Tianjin, China) and the National Institute for the Control of Pharmaceutical and Biological Products (Beijing, China), respectively.

### 2.2. Animals and Sample Preparation

BALB/c female mice (6–8 weeks old, 16–18 g) supplied by LCBL (Liaoning Changsheng Biotechnology Limited by Share Ltd., Benxi, China) were housed in cages measuring 25 cm × 18 cm × 13 cm and maintained under controlled temperature conditions of 23 ± 1 °C with a light/dark cycle of 12 h:12 h. They were given a standard rodent animal diet (commercial food pellets) and drinking water ad libitum for one week prior to the experiments, after which they underwent a fasting period of twelve hours. The experiments were conducted at consistent times (08:00 a.m. and 15:00 p.m.) to eliminate any variations due to time of day.

In total, 32 mice were randomly assigned to four groups, with 8 in each group: control group (Con), infection group (IF), infection group treated with 3 mg/kg·d IOG (IFL_3_), and infection group treated with 6 mg/kg·d IOG (IFL_6_).

*R. ornithinolytica* B1645-1 was cultured in Mueller–Hinton liquid broth added with 0.5 × minimal inhibitory concentration (MIC) IPM (MIC > 8 µg/mL) at 37 °C with 200 rpm until the OD_600_ of 1.5–2.0. First, all of the IF, IFL_3_, and IFL_6_ mice were administrated bacterial solution [350 µL, 1.0×10^10^ colony-forming units (CFU)/mL] viagastric intubation for 24 h. Subsequently, the IFL_3_ and IFL_6_ groups received IOG at doses of 3 mg/kg·d and 6 mg/kg·d (200 µL) for five consecutive days, respectively. The Con was given an equivalent volume of 0.9% normal saline under identical conditions. At both the 24 h and 120 h time points after administration of IOG, the mice were euthanized. Fresh transverse colon contents (approximately 0.1 g) measuring approximately 1 cm in length were dissected and flash-frozen in liquid nitrogen for 30 s before being stored at −80 °C until DNA extraction for microbial diversity and *bla*_NDM-1_ gene analysis.

All experiments using bacteria, IPM, IOG, and mice were performed at the Medical Center of Hubei University of Medicine (Licence No.: SYXK 2017-0093). The animal experiments were conducted upon approval from the Ethical Committee for Vertebrate Experiments of Hubei University of Medicine (Ethical approval No. 2020-086).

### 2.3. DNA Extraction, PCR Amplification, and Sequencing

Under ice-water bath conditions, 0.1 g of the sample was immersed in 1 mL of sterile phosphate-buffered saline (PBS) (0.05 M, pH 7.4), vigorously vortexed, and then centrifuged at 200 rpm for 5 min to remove any remaining coarse particles. After this procedure was repeated three times, the combined supernatant was used to precipitate bacteria at 10,000–12,000 rpm for 10 min.

The E.Z.N.A.^®^ soil DNA kit (Omega) was utilized to extract DNA from the contents of mouse transverse colon. The V3–V4 regions of 16S rDNA were amplified with primers 338F (5′-ACTCCTACGGGAGGCAGCAG-3′) and 806R (5′-GGACTACHVGGGTWTCTA AT-3′). The amplification reaction was as follows: 95 °C pre-degeneration for 3 min, 95 °C degeneration for 30 s, 55 °C annealing for 30 s, and 72 °C extension for 30 s, for a total of 27 cycles, and then extension at 72 °C for 10 min. The raw sequence data were submitted to the NCBI’s Short Read Archive (SRA) (BioProject ID: PRJNA898665).

### 2.4. Microbial Diversity Analysis

The sequence data were processed using Quantitative Insights Into Microbial Ecology (QIIME2; v2022.2) [28]. Paired-end sequences were aligned using the R software (version 4.1) and processed using the Divisive Amplicon Denoising Algorithm 2 (DADA2) version 1.20.0 workflow, followed by mapping to amplicon sequence variants (ASVs) for those with >100% pairwise identity [28,29]. ASVs were taxonomically classified using QIIME with the SILVA reference database (version 138) to construct a phylogenetic tree through the IQ-TREE [30]. *Alpha* diversity was used to assess species richness within group based on the Chao, ACE, Shannon, and Simpson indices. *Beta* diversity was performed to measure differences in abundance between groups with non-metric multidimensional scaling (NMDS) at the ASV level [31]. Linear discriminant analysis effect size (LEfSe) was used for multi-level species difference discriminant analysis, with a logarithmic linear discriminant analysis (LDA) score > 2 and *p* < 0.05 considered significant [32].

### 2.5. Screenation and Identification of Strains Carrying Bla_NDM-1_ Gene

**Figure 1:**
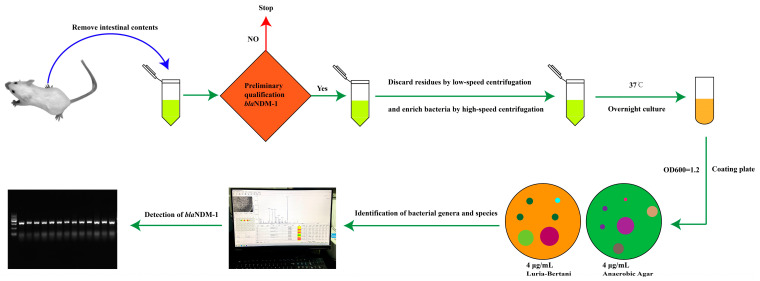
The screening process of *bla*_NDM-1_-positive bacteria.

Based on the operation flow shown in Figure 1, 0.1 g of mouse transverse colon content was mixed in 1 mL of sterile saline and centrifuged at 4000 rpm for 2 min. After three repetitions, the combined supernatant was centrifuged at 12,000 rpm for 2 min at 4 °C to enrich bacteria. The bacteria were inoculated into Luria–Bertani liquid medium (10 g/L NaCl, 10 g/L Tryptone, and 5 g/L Yeast Extract) without antibiotics and routinely cultured overnight at 37 °C. Then, 100 μL of the suspension diluted with saline was coated on LB agar medium (10 g/L NaCl, 10 g/L Tryptone, 5 g/L Yeast Extract, and 15 g/L Agar) containing IPM (4 μg/mL) at 37 °C for 48 h to count CFUs. In the same way, anaerobic agar medium (Pancreatic Casein Peptone 20 g/L, Sodium Chloride 5 g/L, Sodium Formaldehyde Sulfite 1 g/L, Water-Soluble Aniline Blue 0.002 g/L, Glucose 10 g/L, Agar 20 g/L, and Sodium Thioglycolate 2 g/L) with 4 μg/mL IPM was used to separate anaerobic bacteria from the samples [33]. CFUs from plates containing IPM were considered as *bla*_NDM-1_-positive colonies.

To determine the accuracy of *bla*_NDM-1_-positive-colony screening results, PCR was used to detect the *bla*_NDM-1_ gene. Amplification was conducted with the primer set F(5′-CTCGCACCGAATGTCTGGC-3′) and R(5′-GGGGCGTAGTGCTCAGTGTC-3′). The PCR amplification reactions included 25 µL of 2 × NovoStar Green PCR Mix, 2 µL each of upstream and downstream primers (1 mM), 1 µL of DNA template, and ddH_2_O added to a total volume of 50 µL. The PCR conditions were as follows: pre-denaturation at 95 °C for 5 min, denaturation at 95 °C for 30 s, annealing at 55 °C for 30 s, and extension at 72 °C for 1 min, for a total of 34 cycles, and then extension at 72 °C for 5 min. The amplified products were separated via 1% agarose gel electrophoresis (110 V, 30 min) and sequenced. The gene sequence was compared with the GenBank database to determine the *bla*_NDM-1_ gene, and the bacterial strains carrying *bla*_NDM-1_ were identified using mass spectrometry method [34].

### 2.6. Statistical Analysis

One-way ANOVA with Tukey’s multiple comparison test and Kruskal–Wallis H test were used to compare the data among groups, and *p* < 0.05 was considered to be statistically significant [35]. MetaPhlAn2 was used to determine species abundances [36]. GraphPad Prism v8.0 and R package were used to generate graphs for this study.

## 3. Results 

### 3.1. The Diversity of Intestinal Microbiota

A total of 3,829,368 sequences (>202 bp) were derived from 32 samples. After DADA2 noise reduction, 1,541,391 effective sequences were retained and 10,585 ASV sequences were clustered according to the minimum number of sample sequences (16,326 sequences) (due to the death of one mouse and the lack of sequences for two mice, three inefficient samples were eliminated) (Table S1). The rarefaction curves of various samples reached a plateau, indicating that the sampling was effective and the ASV datasets were successfully recovered (Figure S1).

**Figure 2:**
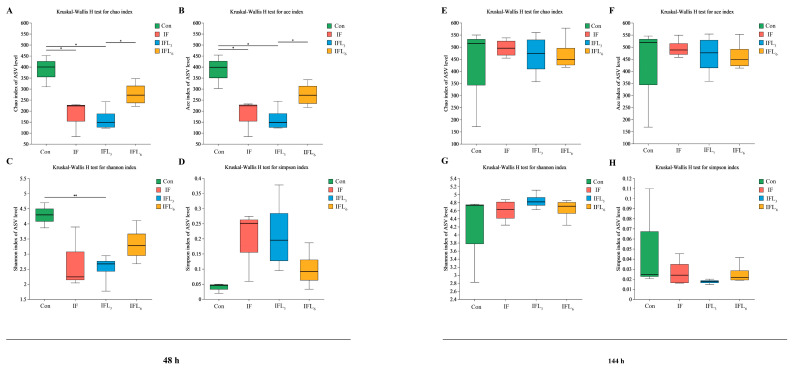
The indices of *Alpha* diversity (Chao, ACE, Shannon, and Simpson) within four groups at 48 h and 144 h. (**A**,**E**) Chao and (**B**,**F**) ACE indices were used to determine species richness; (**C**,**G**) Shannon and (**D**,**H**) Simpson indices were used to estimate bacterial diversity. Significant differences are denoted by asterisks according to the *t*-test (*: *p* < 0.05; **: *p <* 0.01).

The Chao and ACE indices were used to determine species richness, while the Simpson and Shannon indices were used to estimate bacterial diversity (Figure 2). At 48 h, significant differences in species richness estimated based on the Chao and ACE indices were observed among the four groups, as demonstrated by Figure 2A,B. After infection, the species richness in the IF group was significantly lower than the Con group (*p* < 0.05) (Figure 2A), and the species richness was not recovered until the dosage of IOG reached 6 mg/kg·d (IFL_6_) (Figure 2B). The analysis of bacterial diversity estimated based on the Simpson and Shannon indices revealed variations among the different life stages, although these differences did not reach statistical significance (Figure 2C,D). No significant difference in species richness (Figure 2E,F) or diversity (Figure 2G,H) was observed among the four groups at 144 h (*p* > 0.05).

**Figure 3:**
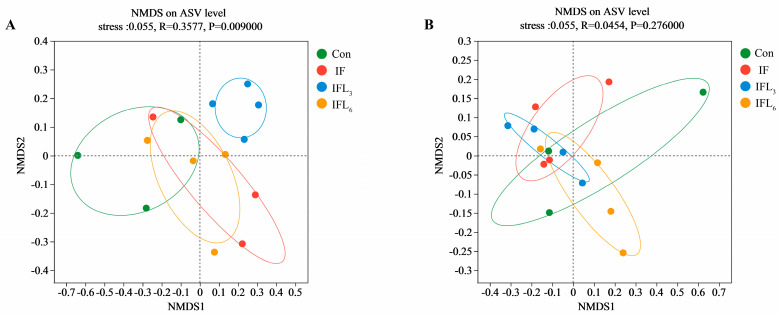
The indices of *Beta* diversity between four groups at 48 h and 144 h. The non-metric multidimensional scaling (NMDS) analyses on amplicon sequence variant (ASV) level at 48 h (**A**) and 144 h (**B**) are shown. The stress value represents the fitting degree of NMDS analysis (stress < 0.05, excellent fitting; stress < 0.1, good fitting; stress < 0.2, average fitting; stress > 0.3, poor fitting). Analysis of similarities (ANOSIM) (R: −1−1, R value close to 1 indicates agreater difference between groups) was used to test the difference between groups.

Figure 3 shows that NMDS had a better fitting degree in ASV levels at 48 h and 144 h (stress = 0.055). The difference between groups at 48 h was greater than the difference within groups (R = 0.3577, *p* = 0.009). There were significant differences in community composition between groups (*p* < 0.05), indicating that all four groups were effective (Figure 3A). However, at 144 h, there was no significant difference observed between the Con, IF, IFL_3_, and IFL_6_ groups (*p* > 0.05), indicating that IOG had no further impact on the community (Figure 3B).

### 3.2. Taxonomic Comparison of Intestinal Microbiota

**Figure 4:**
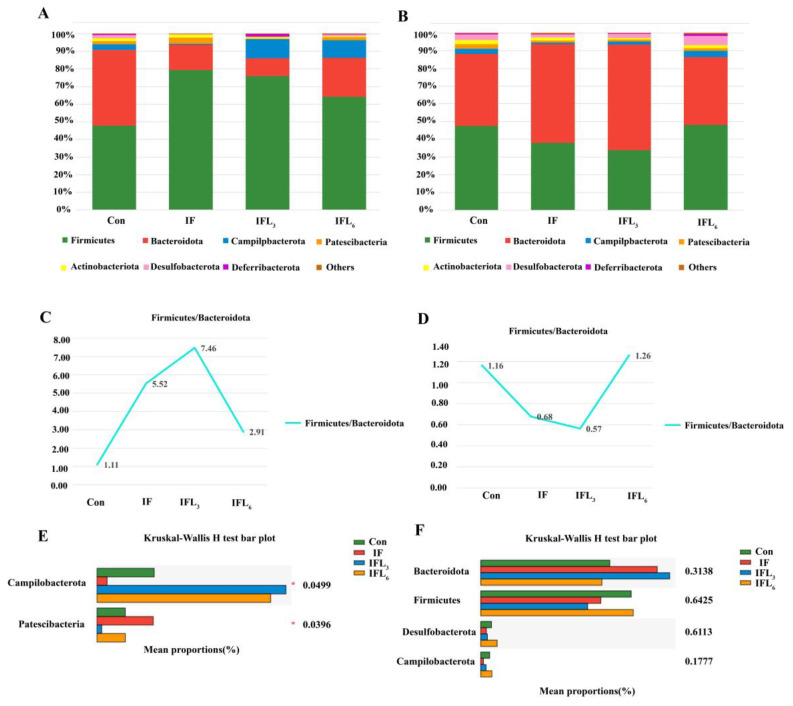
The relative abundance of bacterial phyla in the intestinal microbiota of mice. Only taxa with a relative abundance ≥ 1% in at least one sample were analyzed at 48 h (**A**) and 144 h (**B**). (**C**,**D**) represents the *Firmicutes*/*Bacteroidota* ratio at 48 h and 144 h, respectively. (**E**) Phyla with significant differences at 48 h. (**F**) Phyla with significant differences at 144 h. The seven main phyla of intestinal microbiota include *Firmicutes* (green), *Bacteroidota* (red), *Campilobacterota* (blue), *Desulfobacterota* (pink), *Patescibacteria* (orange), *Actinobacteriota* (yellow), *Deferribacterota* (purple), and others (species abundance < 0.01) (brown). The phylum is represented by a bar graph, and the abscissa represents the ASV level of the phylum.

The compositions of the intestinal microbiota at the phylum and genus levels were characterized. The community barplot analysis showed that the microbiota of 29 samples mainly covered seven phyla, including *Firmicutes*, *Bacteroidota*, *Campilobacterota*, *Desulfobacterota*, *Patescibacteria*, *Actinobacteriota*, and *Deferribacterota* (Figure 4A,B), of which *Firmicutes* and *Bacteroidota* were the dominant phyla. 

At 48 h (Figure 4A), the dominant phyla in IF were *Firmicutes*, *Bacteroidota*, *Campilobacterota*, and *Patescibacteria*, with the relative abundances of 79.45%, 14.39%, 0.58% and 3.24%, respectively, while those of Con were 47.81%, 43.01%, 3.29%, and 1.63%. Surprisingly, IOG contributed to significant changes in intestinal microbiota in the IFL_3_ and IFL_6_ groups. The mice in the IFL_3_ group demonstrated a different intestinal microbiota pattern, consisting of 75.99% *Firmicutes*, 10.19% *Bacteroidota*, 10.88% *Campilobacterota*, and 0.27% *Patescibacteria*. In particular, the IFL_6_ group showed a constantly changing pattern of intestinal microbiota, including 64.24% *Firmicutes*, 22.11% *Bacteroidota*, 10% *Campilobacterota*, and 1.63% *Patescibacteria*. The *Firmicutes*-to-*Bacteroidota* ratio in the Con, IF, IFL_3_, and IFL_6_ groups were 1.11, 5.52, 7.46, and 2.91-fold, respectively (Figure 4C). As shown in Figure 4, *Patescibacteria* and *Campilobacterota* are the phyla leading to significant differences between groups (*p* < 0.05) (Figure 4E). At 144 h (Figure 4B), the relative abundance of *Firmicutes* decreased significantly (40%, 40%, and 50%, respectively) in the IF, IFL_3_, and IFL_6_ groups, while the relative abundance of *Bacteroidota* increased significantly (55.71%, 59.65%, and 38.22%, respectively). The *Firmicutes*/*Bacteroidota* ratio in the Con, IF, IFL_3_, and IFL_6_ groups were 1.16, 0.68, 0.57, and 1.26-fold, respectively (Figure 4D). There was no significant difference between groups in terms of *Bacteroidota*, *Firmicutes*, or other phyla (*p* > 0.05) (Figure 4F).

**Figure 5:**
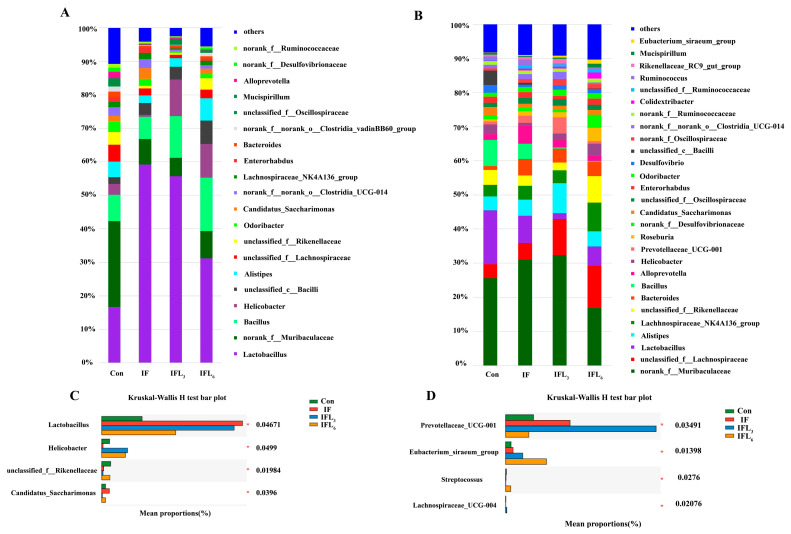
The relative abundance of the bacterial genera in the intestinal microbiota of mice. Only taxa with a relative abundance ≥1% in at least one sample were analyzed at 48 h (**A,C**) and 144 h (**B,D**).

**Figure 6:**
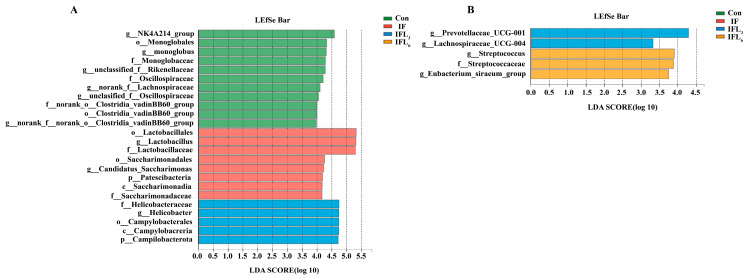
Linear discriminant analysis effect size (LEfSe) at the genus level in mice between Con, IF, IFL_3_, and IFL_6_ groups at 48 h (**A**) and 144 h (**B**). LEfSe, linear discriminant analysis effect size; LEfSe scores > 2 are shown. The prefixes for taxonomic ranks are represented as follows: “p” for phylum, “c” for class, “o” for order, “f” for family, and “g” for genus.

*Norank_f__Muribaculaceae* and *Lactobacillus* were clearly the dominant genera in the Con group (25.46% and 16.68%). At 48 h (Figure 5A), the IF group showed a significant increase of 59.23% in the relative abundance of *Lactobacillus* (*p* < 0.05). Although there was a decreasing trend in the relative abundance of *norank_f__Muribaculaceae* in the IF group, this difference did not reach statistical significance (*p* > 0.05). Following the administration of IOG, the relative abundance of *Lactobacillus* significantly decreased from 55.72% in the IFL_3_ group to 31.13% in the IFL_6_ group, and when the dosage was 6 mg/kg·d (IFL_6_), the relative abundance of *Lactobacillus* recovered to a level with no significant difference compared to the Con group (16.68%). This result suggested that IOG possesses the potential to reduce the increase in *Lactobacillus* count due to infection with *R. ornithinolytica* B1645-1. The relative abundance of *Lactobacillus*, *Helicobacter*, *unclassified_f__Rikenellaceae*, *Candidatus_Saccharimonas*, *norank_f__Lachnospiraceae*, and *Skermanella* exhibited significant differences between groups (*p* < 0.05) (Figure 5C). The LEfSe analysis showed that *Lactobacillus* species had the greatest effect in the IF, IFL_3_, and IFL_6_ groups (Figure 6A).

As shown in Figure 5B (at 144 h), the relative abundance of *unclassified_f__Lachnospiraceae* in the IF, IFL_3_, and IFL_6_ groups increased gradually, while that of *Lactobacillus* significantly decreased to 7.93%, 1.7%, and 5.66%, respectively (15.79% in Con). Figure 5D shows that *Prevotellaceae_UCG-001*, *Eubacterium_siraeum_group*, *Streptococcus*, and *Lachnospiraceae_UCG-004* were significantly different species (*p* < 0.05). The LEfSe analysis also showed that *Prevotellaceae_UCG-001*species and *Streptococcus* species had the greatest impact in the IFL_3_ and IFL_6_ groups, respectively (Figure 6B).

### 3.3. Transmission of Bla_NDM-1_ Gene in Intestinal Microbiota

#### 3.3.1. Screening of Imipenem-Resistant Strains

**Figure 7:**
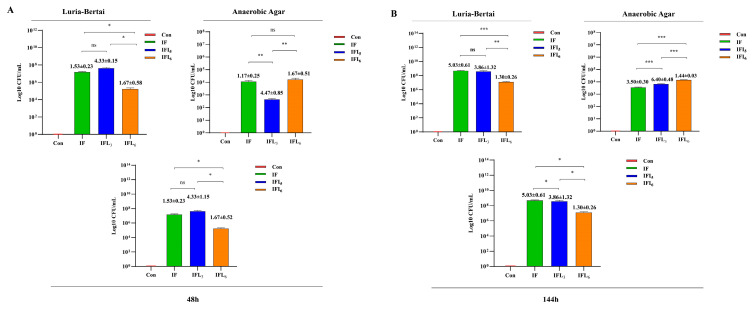
Colony counts of imipenem (IPM)-resistant bacteria in intestinal microbiota. (**A**,**B**) represent the number of bacteria screened using Luria–Bertani agar medium and anaerobic agar medium containing 4 μg/mL IPM at 48 h and 144 h, respectively. *: *p* < 0.05; **: *p <* 0.01; ***: *p <* 0.001; ns: *p >* 0.05.

Drug-resistant strains were screened using Luria–Bertani agar medium and anaerobic agar medium supplemented with IPM at a concentration of 4 μg/mL. On Luria–Bertani agar medium, Figure 7A demonstrates a significant increase in bacterial numbers at 48 h for the IF, IFL_3_, and IFL_6_ groups compared to the Con group, with values of 1.53 ± 0.23 × 10^7^ CFU/mL, 4.33 ± 1.15 × 10^7^ CFU/mL, and 1.67 ± 0.52 × 10^5^ CFU/mL, respectively. Meanwhile, the bacteria count in the IFL_6_ group was significantly lower than that in both the IF and IFL_3_ groups (*p* < 0.05 for both comparisons). Figure 7B also shows that the bacterial counts in the IF, IFL_3_, and IFL_6_ groups increased significantly at 144 h compared to at 48 h, which were 5.03 ± 0.61 × 10^8^ CFU/mL, 3.86 ± 1.32 × 10^8^ CFU/mL, and 1.30 ± 0.26 × 10^7^ CFU/mL, respectively. The bacterial count in the IFL_6_ group was still significantly lower than the counts in the IF and IFL_3_ groups (*p* < 0.001 and *p* < 0.01). On anaerobic agar medium, the number of anaerobic bacteria in the IF, IFL_3_, and IFL_6_ groups increased significantly at 48 h, reaching 1.17 ± 0.25 × 10^4^ CFU/mL, 4.47 ± 0.85 × 10^2^ CFU/mL, and 1.67 ± 0.51 × 10^4^ CFU/mL, respectively. In addition, we also saw that at 144 h, the number of anaerobic bacteria in the IF, IFL_3_, and IFL_6_ groups was 3.53 ± 0.3 × 10^3^ CFU/mL, 6.4 ± 0.48 × 10^3^ CFU/mL, and 1.44 ± 0.03 × 10^4^ CFU/mL, respectively. Combined with the number of colonies in the two culture media, the number of bacteria in the IF, IFL_3_, and IFL_6_ groups at 48 h was 1.53 ± 0.3 × 10^3^ CFU/mL, 4.33 ± 0.3 × 10^3^ CFU/mL, and 1.67 ± 0.3 × 10^3^ CFU/mL, respectively. The bacterial numbers at 144 h were 5.03 ± 0.61 × 10^8^ CFU/mL, 3.86 ± 1.32 × 10^8^ CFU/mL, and 1.30 ± 0.26 × 10^7^ CFU/mL, respectively.

In conclusion, with the extension of infection time, the number of aerobic and anaerobic resistant bacteria significantly increased. However, the number in the IFL_6_ group remained consistently lower than that in the IF and IFL_3_ groups, indicating IOG’s inhibitory effect on the production of drug-resistant bacteria.

#### 3.3.2. Identification of Strains Carrying Bla_NDM-1_ Gene

At 48 h (Table 1), seven genera and eight species of *bla*_NDM-1_-positive bacteria were successfully isolated from the IF, IFL_3_, and IFL_6_ groups, including *Lactobacillus johnsonii*, *Enterococcus faecalis*, *Enterococcus gallinarum*, *Klebsiella pneumoniae*, *Escherichia coli*, *Brevibacterium linens*, *Acinetobacter baumannii*,and *Bacillus pumilus*, of which *Enterococcus* was the dominant genus. However, at 144 h, there were still *bla*_NDM-1_-positive bacteria in five genera (*Enterococcus, Chromobacterium*, *Lactobacillus*, *Microbacterium*, and *Escherichia*) in the IF, IFL_3_, and IFL_6_ groups (Table 1). Except for the Con group, *Lactobacillus* was the dominant genus in all groups, with a total of four species, including *L. johnsonii*, *L. reuteri*, *L. murinus*, and *L. gasseri*.

## 4. Discussion

Enteropathogenic infections and infection-mediated inflammatory responses can disrupt the intestinal ecosystem, including changing the composition and number of intestinal microbiota [16]. *R. ornithinolytica* B1645-1 isolated from the blood sample of a renal transplant patient who died of sepsis was found to be pathogenic and significantly disrupted the composition of intestinal microbiota in mice. Specifically, the *Firmicutes*/*Bacteroidota* ratio was significantly higher in the IF group (5.52:1) than in the Con group (1.1:1). Additionally, at 48 h, there was a notable increase in *Lactobacillus* abundance from 17% in the Con group to 60% in the IF group. During the initial phase (48 h), total colonic bacterial numbers (ASVs) decreased significantly while *Lactobacillus* increased, which may be attributed to pathogenic bacterial colonization and subsequent elimination of *Bacteroidota* [16]. However, the *Firmicutes*/*Bacteroidota* ratio recovered to 0.68:1 at 144 h and *Lactobacillus* was no longer the factor that affected the difference (Figure 6B), indicating that colonizing the intestine by *R. ornithinolytica* alone was not enough to continuously reduce the overall level of intestinal microbiota and alter the proportion of resident bacteria. The effect of *R. ornithinolytica* B1645-1 on the intestinal microbiota of mice was also evident in alterations to other bacteria, such as *norank_f__Muribaculaceae* and *Bacillus* (Figure 5A,B, Table S2).

The major groups of mammalian intestinal microbiota include *Lactobacilli*, *Bifidobacteria*, *Enterobacteria*, *Enterococci*, and *Bacteroides* species [11]. In animals, *Firmicutes* and *Bacteroidota* (such as *Bacteroides* and *Bifidobacteria*), which are strictly anaerobic bacteria, maintain a relatively low level of facultative anaerobes (such as *Enterobacteriaceae* and *Enterococci*), while *Lactobacilli* are abundant in rats and mice [11,15]. IOG treatment for 24 h significantly altered the composition of intestinal microbiota in all groups. The *Firmicutes*/*Bacteroidota* ratio was significantly lower in the IFL_6_ group (2.91:1) compared to the IFL_3_ (7.46:1) and IF (5.52:1) groups, indicating that 6 mg/kg·d IOG had a stronger regulatory effect on intestinal microbiota recovery in infected mice than in other groups. Contrary to previous reports suggesting that IOG promotes an increase in *Lactobacillus* within the intestinal flora of healthy rodents [12], our study surprisingly found a decrease in *Lactobacillus* numbers from 50% in the IFL_3_ group to 30% in the IFL_6_ group at 48 h. This regulatory effect was even more significant at 144 h, indicating that IOG primarily targets *Lactobacillus* as one of the main influencers on intestinal microbiota.

The spread of carbapenemase (KPC, NDM, OXA-48, and VIM)-producing enterobacteria (CPE) clones among patients and the transfer of carbapenemase-encoding genes between enterobacteria in individual patients’ intestinal microbiota have shaped the epidemiological characteristics of CPE [23,37]. However, the transfer of carbapenemase-encoding genes within the intestinal microbiota of individual patients remains poorly understood. The ability of IOG to modulate the intestinal microbiota has been demonstrated through alterations in phyla (*Bacteroidota* and *Firmicutes*) and genera (*Lactobacillus*, *Bacillus*, etc.), which are associated with the transmission of drug-resistant genes within intestinal microbiota. After 48 h of infection, the *bla*_NDM-1_ gene carried by *R. ornithinolytica* B1645-1 was detected in various intestinal bacteria, including *E. faecalis*, *E. gallinarum*, *E. coli*, *K. pneumoniae*, *A. baumannii*, *B. pumilus*, *B.linens*, and *Lactobacillus johnsonii*, with *Enterococcus* being the predominant genus. However, after a 120 h treatment with IOG, intestinal bacteria carrying the *bla*_NDM-1_ gene underwent significant changes and *Lactobacillus* (*L. johnsonii*, *L. reuteri*, *L. murinus*, and *L. gasseri*) became the dominant carrier of the *bla*_NDM-1_ gene. This finding indicated that the *bla*_NDM-1_ gene had been transferred from pathogenic bacteria (*R. ornithinolytica* B1645-1) to *Enterococcus* within 48 h, and subsequently to *Lactobacillus* within 144 h, either through natural recovery in the IF group or after treatment with IOG in the IFL_3_ and IFL_6_ groups. Although IOG exhibited efficacy in reducing the number of bacterial genera or species carrying drug-resistant genes and the abundance of drug-resistant bacteria (Figure 7 and Table 1), *Lactobacillus*, as a probiotic with intestinal advantages [38], has become the carrier of drug-resistant genes and is at risk of expanding the transmission of drug-resistant genes.

*R. ornithinolytica* B1645-1 induced a shift in the microbial composition, leading to ecological disorder. This finding has significant implications for comprehending the biological function of *R. ornithinolytica* and for clinical prevention and treatment. IOG rectified this interference and facilitated the translocation of the *bla*_NDM-1_ gene from *Enterococcus* to *Lactobacillus*, providing strong support for its clinical application. However, as a recipient for resistant genes, *Lactobacillus* warrants further investigation.

## 5. Conclusions

*R. ornithinolytica* administered orally is sufficient to perturb the composition of intestinal microbiota and facilitate the transfer of the *bla*_NDM-1_ gene in intestinal microbiota. Luteolin-7-O-glucoside can modulate this disordered intestinal microbiota, inhibit the production of drug-resistant bacteria, and promote the transfer of the *bla*_NDM-1_ gene from the genus *Enterococcus* to *Lactobacillus*. However, *Lactobacillus* should be paid more attention as the recipient of drug-resistant genes because it may be an inevitable donor.

## Figures and Tables

**Table 1:** The *bla*_NDM-1_-positive bacteria in intestinal microbiota.

Time	Groups	IPM	Species	
			Luria–Bertani Agar	Anaerobic Agar
48 h	Con	4 μg/mL	----	----
	IF		*Enterococcus faecalis*, *E. gallinarum*, *Acinetobacter baumannii*, *Klebsiella pneumoniae*	----
	IFL_3_		*E. faecalis*, *E. gallinarum*, *Escherichia coli*, *Bacillus pumilus*, *Brevibacterium linens*, *K. pneumoniae*	*Lactobacillus johnsonii*
	IFL_6_		*E. faecalis*, *E. gallinarum* *E. coli*, *K. pneumoniae*	----
144 h	Con	4 μg/mL	----	----
	IF		*E. faecalis*, *Chromobacterium violaceum*	*L. johnsonii*, *L. murinus*, *L. reuteri*
	IFL_3_		*Microbacterium* sp., *E. coli*	*L. johnsonii*, *L. gasseri*, *L. murinus*, *L. reuteri*
	IFL_6_		*E. faecalis*	*L. gasseri*, *L. johnsonii*, *L. reuteri*

## Data Availability

The raw data for 16S rRNA gene sequences presented in this study are openly available in the NCBI BioProject database, reference number [PRJNA898665].

## Supplementary Materials

The following supporting information can be downloaded at https://www.mdpi.com/article/10.3390/microorganisms11102477/s1, Figure S1: Rarefaction curves for sobs index (A) and Shannon index (B) at 48 h. Table S1: Information of the original sequences, optimized sequences, DADA2 noise reduction, and ASVs. Table S2: Relative abundances of the bacterial phyla in the intestinal microbiota of the mice at 48 h.
