# Supplementary figures and images for: Effects of Luteolin-7-O-Glucoside on Intestinal Microbiota Dysbiosis and Drug Resistance Transmission Caused by Raoultella ornithinolytica B1645-1: Modulating the Composition of Intestinal Microbiota and Promoting the Transfer of blaNDM-1 Gene from Genus Enterococcus to Lactobacillus in Mice
Source: Microorganisms. 2023 Oct 2;11(10):2477. doi: 10.3390/microorganisms11102477 (PMC10609467; doi:10.3390/microorganisms11102477)

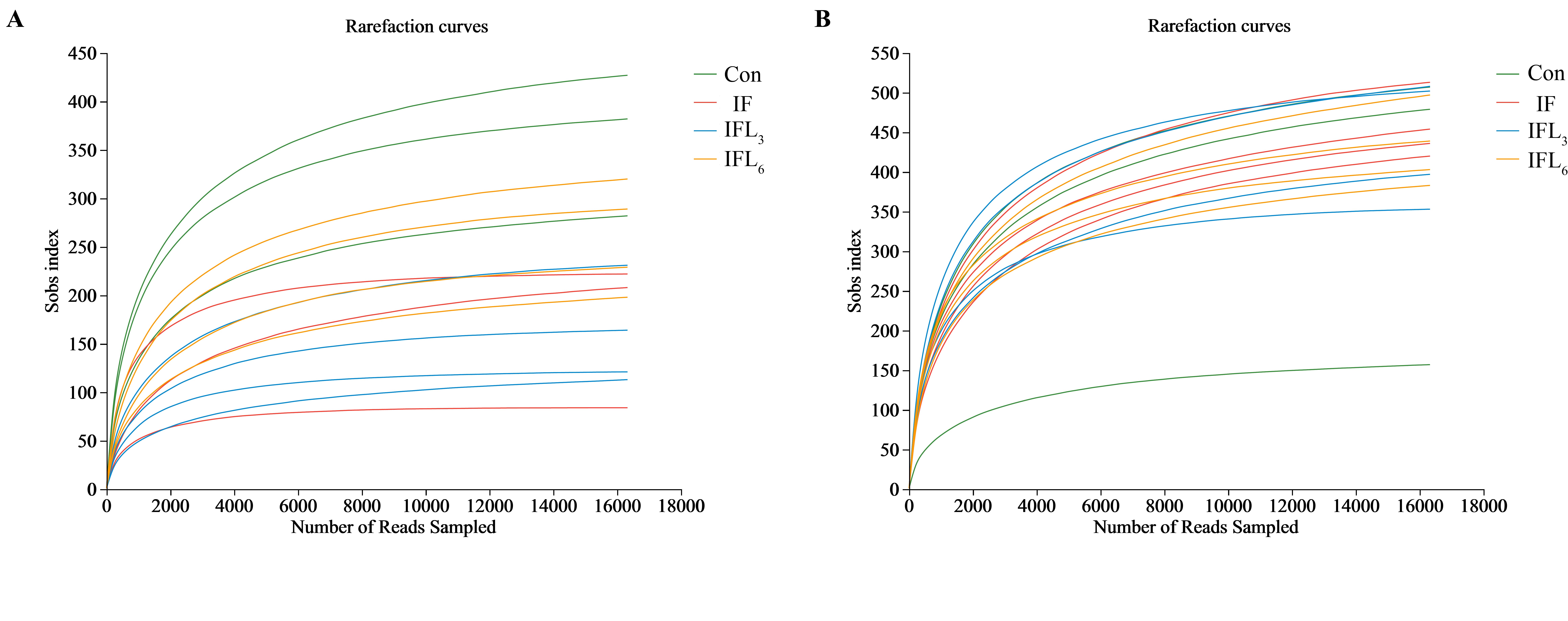

Supplement: Supplementary file 1 [file microorganisms-11-02477-s001.zip › Supplementary Files/Fig S1.jpg]
